# Beyond *Saccharomyces pastorianus* for modern lager brews: Exploring non-*cerevisiae Saccharomyces* hybrids with heterotic maltotriose consumption and novel aroma profile

**DOI:** 10.3389/fmicb.2022.1025132

**Published:** 2022-11-10

**Authors:** Nikola Y. Gyurchev, Ángela Coral-Medina, Susan M. Weening, Salwa Almayouf, Niels G. A. Kuijpers, Elke Nevoigt, Edward J. Louis

**Affiliations:** ^1^Centre of Genetic Architecture of Complex Traits, Department of Genetics and Genome Biology, University of Leicester, Leicester, United Kingdom; ^2^School of Science, Jacobs University Bremen, Bremen, Germany; ^3^SPO, Université de Montpellier, INRAE, Institut Agro, Montpellier, France; ^4^School of Microbiology, University College Cork, Cork, Ireland; ^5^Department of Biotechnology, Delft University of Technology, Delft, Netherlands; ^6^Research and Development, HEINEKEN Supply Chain B.V, Zoeterwoude, Netherlands

**Keywords:** lager brewing, yeast, *de novo* hybrids, diversity, maltotriose utilization, heterosis, novel aroma profiles, non-*cerevisiae*

## Abstract

Non-domesticated, wild *Saccharomyces* yeasts have promising characteristics for beer diversification, particularly when used in the generation of *de novo* interspecific hybrids. A major motivation for the current work was the question whether attractive novel *Saccharomyces* interspecific hybrids can be created for the production of exotic lager beers without using the genomic resources of the ale yeast *Saccharomyces cerevisiae*. Importantly, maltotriose utilization is an essential characteristic typically associated with domesticated ale/lager brewing strains. A high-throughput screening on nearly 200 strains representing all eight species of the *Saccharomyces* genus was conducted. Three *Saccharomyces mikatae* strains were able to aerobically grow on maltotriose as the sole carbon source, a trait until recently unidentified for this species. Our screening also confirmed the recently reported maltotriose utilization of the *S. jurei* strain D5095^T^. Remarkably, *de novo* hybrids between a maltotriose-utilizing *S. mikatae* or *S. jurei* strain and the maltotriose-negative *Saccharomyces eubayanus* strain CBS 12357^T^ displayed heterosis and outperformed both parents with regard to aerobically utilizing maltotriose as the sole source of carbon. Indeed, the maximum specific growth rates on this sugar were comparable to the well-known industrial strain, *Saccharomyces pastorianus* CBS 1513. In lager brewing settings (oxygen-limited), the new hybrids were able to ferment maltose, while maltotriose was not metabolized. Favorable fruity esters were produced, demonstrating that the novel hybrids have the potential to add to the diversity of lager brewing.

## Introduction

Brewing has been connected to human activity for millennia. The long-term unintentional domestication of baker’s yeast (mainly ale yeasts of the species *Saccharomyces cerevisiae*) has developed its superior fermentation capabilities and the pleasant mixture of aroma and flavor compounds in ale-style beers. Moreover, standardizing and regulating the brewing process in the medieval times to be conducted at lower temperatures (*ca.* 12°C) gave rise to lager-style beers with their distinguished clean, crisp, and refreshing character (Hornsey, 2003). The yeast responsible for lager fermentation was identified to be different from *S. cerevisiae* and named *Saccharomyces pastorianus*, a hybrid between an ale *S. cerevisiae* and the cold-tolerant *Saccharomyces eubayanus*. The latter parental wild yeast has only been discovered a decade ago (Libkind et al., 2011). *S. eubayanus* strains have been isolated from different regions of the world (Libkind et al., 2011; Bing et al., 2014; Peris et al., 2014; Gayevskiy and Goddard, 2016; Nespolo et al., 2020). Two of those isolates have also been used by the brewing industry to brew novel types of lager beer (Lovin Dublin, 2018).

Lager beer brewed with the hybrid yeast *S. pastorianus* makes up the majority of global beer volume; however, preferences toward novel specialty beers with exotic aroma profiles led to the exponential increase of craft breweries (Garavaglia and Swinnen, 2017; Jaeger et al., 2020). Industrial *S. pastorianus* strains are genetically related and therefore exhibit limited phenotypic diversity including a narrow range of flavor/aromatic profiles (Dunn and Sherlock, 2008; Okuno et al., 2015; Gallone et al., 2019; Salazar et al., 2019). Therefore, there has been a high interest in increasing aromatic complexity in lager beer by exploiting the biodiversity of wild yeasts of the *Saccharomyces* clade either individually or after hybridization with other species at the relative cold fermentation temperatures. Indeed, interspecific hybridization techniques have been a powerful tool in this context, particularly since hybrids could outperform both parental species due to heterosis effects as previously demonstrated for *de novo S. cerevisiae* × *S. eubayanus* crosses (Hebly et al., 2015; Krogerus et al., 2015; Mertens et al., 2015). Such a novel hybrid with enhanced brewing and aromatic properties has recently been commercialized for the production of lager beer (Lallemand Brewing, 2022).

The *Saccharomyces* clade currently comprises eight recognized species from various sources and locations: *S. cerevisiae*, *S. paradoxus* (Batshinskaya, 1914), *S. mikatae* (Naumov et al., 2000), *S. eubayanus* (Libkind et al., 2011), *S. kudriavzevii* (Naumov et al., 2000), *S. uvarum* (Beijerinck, 1898), *S. arboricola* (Wang and Bai, 2008), and the most recently identified *S. jurei* (Naseeb et al., 2017). Non-domesticated (wild) representatives of the genus have already shown their fermentative and desired fruity aroma potential in lager brewing with several examples recently reported for *S. eubayanus* (Eizaguirre et al., 2018; Lovin Dublin, 2018; Mardones et al., 2020), *S. paradoxus* (Nikulin et al., 2020b), and *S. jurei* (Giannakou et al., 2021; Hutzler et al., 2021). Such studies have been commonly restricted to a single or few tested strains.

Alongside the potential benefits for aroma complexity, there are a few typical traits in wild yeasts that are regarded undesired in lager brewing. These include the production of phenolic off-flavors such as 4-vinyl guaiacol (4-VG; Gallone et al., 2016), lack of cold tolerance (Nikulin et al., 2018), suboptimal flocculation (Hutzler et al., 2021), and most importantly, inefficient fermentation of the most abundant sugars in brewer’s wort, maltose and maltotriose (Steensels and Verstrepen, 2014; Methner et al., 2019; Nikulin et al., 2020a). Maltotriose is typically utilized at the end of the fermentation (Dietvorst et al., 2005). Residual amounts of the trisaccharide could affect both flavor and ethanol yield, an important economical parameter to produce lagers (Zheng et al., 1994). Maltotriose utilization in yeast is complex, being determined by *MAL* loci harboring three gene families: *MALT* encoding an oligosaccharide permease, *MALS* encoding an α-glucosidase and *MALR* encoding a regulator responsible for the transcriptional induction of *MALT* and *MALS* by maltotriose (Charron et al., 1989; Brouwers et al., 2019b).

In general, non-conventional (wild) yeast species of the *Saccharomyces* clade are thought not to be able to grow on maltotriose as the sole carbon source (Mtt^−^ phenotype). This also holds for the species *S. eubayanus* since all strains known so far are unanimously maltotriose-negative (Mtt^−^) (Mardones et al., 2020). In fact, efficient utilization of maltotriose (Mtt^+^ phenotype) and particularly its fermentation to ethanol was assumed to be generally restricted to ale *S. cerevisiae* and *S. pastorianus* maltotriose-positive (Mtt^+^) strains at the time when the current study was initiated. The ability of the natural hybrid yeast *S. pastorianus* to utilize maltotriose by fermentation was initially attributed solely to the *S. cerevisiae* parent (Hebly et al., 2015; Brickwedde et al., 2018). Later, it was demonstrated that the genome of the Mtt^−^ parent, *S. eubayanus,* contributed as well to this phenotype possibly due to neofunctionalization of the *MAL* genes (Baker and Hittinger, 2019; Brouwers et al., 2019b) or regulatory crosstalk between the two parental genomes (Brouwers et al., 2019a). Interestingly, a few strains of the *S. eubayanus* species including the well-studied CBS 12357^T^ have demonstrated evolvability to maltotriose utilization (Baker and Hittinger, 2019; Brouwers et al., 2019b). These results suggest that *S. eubayanus* might be able to provide the genetic resources for a Mtt^+^ phenotype in novel hybrids even without *S. cerevisiae* as a parent. This approach would be particularly promising when using a second wild-type strain that also brings along certain genetic resources enabling maltotriose utilization.

In this study, high-throughput screening (HTS) for growth on maltotriose as the sole carbon source was applied to a wide range of a *Saccharomyces* isolates and led to the identification of several previously undescribed non-domesticated wild isolates with an Mtt^+^ phenotype. Newly identified wild strains were hybridized with the cold-tolerant *S. eubayanus* CBS 12357^T^ leading to novel non-*cerevisiae Saccharomyces* hybrids. We evaluated the growth performance of the *de novo* hybrids on maltotriose in comparison to the established domesticated *S. pastorianus* CBS 1513 strain. We also tested the potential of the new hybrids to ferment brewer’s wort and brew beer with novel aroma profiles under conditions simulating lager brewing in lab scale.

## Materials and methods

### Strains, plasmids, and media composition

*Saccharomyces* parental strains for the HTS for growth on maltotriose and *S. pastorianus* strains were selected from various geographical locations and origins. The complete list of strains screened for this study is shown in Supplementary Table S1 and the strains selected for further use in hybrids are summarized in Table 1. Yeasts were routinely grown on YPD medium (1% (w/v) yeast extract, 2% (w/v) peptone, and 2% (w/v) glucose) and kept in 25% (w/v) glycerol stocks at −80°C. Selection on Hygromycin B^R^ was performed via the addition of 300 μg mL^−1^ (final concentration).

**Table 1:** List of relevant *Saccharomyces* strains [*S. pastorianus* (*Sc* × *Se*), *S. cerevisiae* (*Sc*), *S. eubayanus* (*Se*), *S. mikatae* (*Sm*), *S. jurei* (*Sj*)] used in this study.

Strain name	Species	Location	Origin	Genotype	Reference	Notes
UWOPS83-883-2	*Sc*	Navassa Island	*Drosophila* sp.	Wild type	UWOPS database	
C6.1	*Sc*	Cameroon	Pam wine	Wild type	Stringini et al. (2009)	
DBVPG 6881	*Sc*	United States	Ridge Winery	Wild type	DBVPG database	
CBS 1462	*Sc* × *Se*	United Kingdom	Brewery	Non-engineered	CBS database	Group I (Saaz)
CBS 1513	*Sc* × *Se*	Denmark	Carlsberg brewery	Non-engineered	CBS database	Group I (Saaz)
CBS 1483	*Sc* × *Se*	Netherlands	Heineken brewery	Non-engineered	CBS database	Group II (Frohberg)
CBS 12357^T^	*Se*	Argentina	Southern beech	Wild type	Libkind et al. (2011)	
NBRC 10997	*Sm*	Japan	Decayed leaf	Wild type	NBRC database	
NBRC 11002	*Sm*	Japan	Decayed leaf	Wild type	NBRC database	
LSYS65-1	*Sm*	China	Bark tree	Wild type	CGMCC database	
D5095^T^	*Sj*	France	Bark tree	Wild type	Naseeb et al. (2017)	
NG1	*Sj*			*ho*::*HPH*	This study	Derived from D5095^T^
NG16	*Sj*			*ho*::*HPH MAT* a	This study	Derived from NG1
NG17	*Sj*			*ho*::*HPH MAT* α	This study	Derived from NG1
Q183	*Se*			*ho*::*HPH MAT* a	Hinks (2018)	Derived from CBS 12357^T^
Q184	*Se*			*ho*::*HPH MAT* α	Hinks (2018)	Derived from CBS 12357^T^
NG87	*Sm*			*ho*::*HPH*	This study	Derived from NBRC 10997
NG88	*Sm*			*ho*::*HPH*	This study	Derived from NBRC 11002
NG89	*Sm*			*ho*::*HPH*	This study	Derived from LSYS65-1
NG90	*Sm*			*ho*::*HPH MAT* a	This study	Derived from NG87
NG91	*Sm*			*ho*::*HPH MAT* α	This study	Derived from NG87
NG92	*Se* × *Sj*			*ho*::*HPH*	This study	Q183 × NG17
NG101	*Se* × *Sm*			*ho*::*HPH*	This study	Q184 × NG90

UWOPS (University of Western Ontario Plant Sciences); CBS (Centraal bureau voor Schimmelcultures); NBRC (Biological Resource Center, NITE); CGMCC (China General Microbiological Culture Collection Center); DBVPG ((Dipartimento di Biologia Vegetale, Università di Perugia) Perugia).

Experiments assessing growth on maltotriose were conducted in either YP medium containing 2% (w/v) maltotriose (YP + Mtt) or synthetic medium (SM) according to Verduyn et al. (1992) with 2% (w/v) maltotriose (SM + Mtt) adjusted to pH 6.5 with 2 M KOH; 2% (w/v) agar was added in solid media. Pre- and intermediate cultures were performed in SM containing 2% (w/v) glucose and cells were washed with SM without carbon source. Wort fermentations were conducted in industrial wort diluted to 12 ^o^P supplemented with 0.6 mg L^−1^ ZnSO_4_.7H_2_O final concentration; sugar content is listed in Supplementary Table S8. To sporulate yeast, potassium acetate (KAc) medium was used containing 2% (w/v) potassium acetate, 0.22% (w/v) yeast extract, and 0.05% (w/v) glucose adjusted at pH 7 with 1 M HCl or 2.5 M NaOH after which 0.087% (w/v) synthetic complete medium powder (Supplementary Table S1) and 2.5% (w/v) agar were added.

### General molecular biology techniques

Primers used in this study are listed and detailed in Supplementary Table S2. PCRs for amplifying deletion cassettes, verification, and species-specific identification were performed using OneTaq® DNA Polymerase (New England BioLabs, Germany) or BIOTAQ™ DNA Polymerase (Bioline, UK). Phusion® High-Fidelity DNA Polymerase (New England BioLabs, Germany) was used to amplify fragments for Gibson assembly according to the respective manufacturer’s protocol. Colony PCR was applied *via* initially suspending cells in 0.02 M NaOH and incubating at 95°C for 10 min and using the supernatant as a DNA template.

Gibson assembly reactions composition and transformation protocols were adapted from the Gibson Assembly® Master Mix instructions (New England BioLabs, Germany) in which 15 μl of Gibson assembly master mix (1.33×; Supplementary Table S1) was used instead of 10 μl and LB medium instead of SOC medium. Transformation of deletion cassettes in *Saccharomyces* was conducted as described in Gietz et al. (1995) except for the use of lower heat shock temperatures – 37°C for 5 and 20 min applied on *S. mikatae* and *S. jurei* strains, respectively.

### Screening of *Saccharomyces* strains for growth on maltotriose

*Saccharomyces* strains were initially tested for growth on maltotriose *via* the phenotyping on solid medium (PHENOS) pipeline from Barton et al. (2018). PHENOS produces reproducible proxy growth curves based on automatic absorbance measurements on the colony thickness subtracting the agar absorbance of a rectangular array plate fitting up to 96 strains with four technical replicates. To unequivocally confirm the species designation of the array glycerol stocks, ITS sequencing was performed (White et al., 1990). Strains from Arrays I and II (Supplementary Table S1) were analyzed for growth on YP + Mtt at 30°C. Strains from array III (Supplementary Table S1) were analyzed for growth on SM + Mtt at 25°C. Maltotriose was initially supplied from Sigma-Aldrich, UK with ≥90% purity and added to the YP solid media. Subsequently to decrease impurities, maltotriose was obtained from Glentham Life Sciences, UK, with ≥95% purity and was used instead for further experiments.

Mtt^+^ candidate strains were tested for growth in liquid SM + Mtt. Initially, 3 ml SM 2% (w/v) glucose pre-cultures were inoculated with single-cell colonies in triplicates in 10 ml glass tubes at 25°C and 250 rpm for ~16 h. Intermediate 3 ml SM 2% (w/v) glucose cultures were prepared by inoculating at 0.2 OD_600_ and incubating at 25°C at 250 rpm for 24 h. Cells were washed once with SM without carbon source and once with SM + Mtt before inoculating the final 0.7 ml SM + Mtt culture at 0.2 OD_600_ with two technical replicates in Krystal 24-well clear bottom microplate (Porvair Sciences, Leatherhead, UK) cultivated in the Growth Profiler 1,152 (Enzyscreen, Netherlands) at 200 rpm and 25°C. The Growth Profiler determines the density of the cultures by measuring pixel density from images taken every 40 min of individual wells correlated to equivalent OD_600_ values *via* a calibration curve with the equation of the best fit line: OD_600_ equivalent = 6.1761.10 × Green value^3.4784^. The calibration curve was based on *S. cerevisiae* CEN.PK113-1A and was applied to generate OD_600_ values for all strains tested for a comparative analysis.

### Hybrid construction and confirmation

Interspecific hybrids were constructed by firstly disrupting the *HO* gene in the parental strains to abolish mating-type switching and self-mating and obtain stable haploids (Strathern et al., 1982). The cassettes to integrate the *HPH* hygromycin marker in *S. jurei* D5095^T^, *S. mikatae* NBRC 10997, *S. mikatae* NBRC 11002, and *S. mikatae* LSYS65-1 were amplified from pAG32 (Goldstein and Mccusker, 1999), pUC18_*ho*::*HPH_*NBRC_10,997, pUC18_*ho*::*HPH*_NBRC_11,002, and pUC18_*ho*::*HPH*_LSYS65-1, respectively, (Supplementary Table S1). Homology arms with 45 bp and 500 bp were applied on *S. jurei* and *S. mikatae* strains, respectively, targeting the beginning of the *HO* open reading frame (ORF) to disrupt it and generate strains NG1, NG87, NG88, and NG89.

Sporulation and tetrad dissection were conducted *via* standard protocols (Sherman and Hicks, 1991) to obtain stable haploids confirmed to be either *MAT* “a” or “α” *via* mating-type PCR (Supplementary Table S2) and halo assay (Julius et al., 1983). Stable haploids were not successfully isolated from NG88 (*S. mikatae* NBRC 11002 *ho*::*HPH*) and NG89 (*S. mikatae* LSYS65-1 *ho*::*HPH*). Interspecific hybrids NG92 (*Se* × *Sj*) and NG101 (*Se* × *Sm*) were constructed *via* mass mating as described by Sherman and Hicks (1991). Successful hybrid formation was confirmed *via* initially re-streaking the cell mass for single colonies three times after which they were sporulated to confirm tetrad formation. The production of spores demonstrated their hybrid nature favoring sporulating hybrids in the verification process. Two and four biological replicates of *Se* × *Sj* and *Se* × *Sm* hybrids, respectively, were successfully isolated. Sporulation efficiency was determined for twenty tetrads per hybrid resulting in ~1% spore viability for each hybrid tested and glycerol stocks were randomly selected and designated as NG92 (*Se* × *Sj*) and NG101 (*Se* × *Sm*). The interspecific hybrid formation was further verified by individual species-specific PCRs (Muir et al., 2011; Reuben et al., 2013; Naseeb et al., 2021).

### Aerobic shake-flask cultivations in synthetic medium with maltotriose as the sole carbon source

Cultivations of 50 ml SM + Mtt in shake flasks were performed in 500 ml Erlenmeyer flasks. Pre- and intermediate cultures and washing steps were conducted as the cultivations in the Growth Profiler upscaled for the intermediate cultures to 10 ml SM 2% (w/v) glucose in 100 ml Erlenmeyer flasks at orbital shaking of 250 rpm at 25°C. Growth was monitored by sampling every 2 h to measure OD_600_ during the exponential growth phase of each strain. The maximum specific growth rate and lag phase duration were calculated for *S. pastorianus* CBS 1513 and the interspecific hybrids NG92 (*Se* × *Sj*) and NG101 (*Se* × *Sm*). Samples for HPLC measurement were taken every 24 h to determine maltotriose consumption. Maltotriose and maltose were measured using an Agilent 1,260 LC equipped with a 1,260 Refractive Index Detector and an Bio-Rad Aminex HPX-42A Column (300 × 7.8 mm, 25 micron) operated at 75°C using MilliQ as eluent at a flow rate of 0.5 ml·min^−1^.

### Wort fermentations and aroma profiling

Pre-cultures of 10 ml YPD in 100 ml Erlenmeyer flasks were inoculated with single-cell colonies in triplicates and incubated at 20°C at 80–120 rpm for 24 h. Intermediate cultures of 30 ml 12 ^o^P wort in 300 ml Erlenmeyer flasks were inoculated at 0.2 OD_600_ and incubated at 20°C at 80–120 rpm for 48 h. The inoculum was calculated for each replicate by counting cells on a Neubauer Hemocytometer diluted with 50 mM EDTA to avoid flocculation and adjusted to 1.2 × 10^7 cells/mL. Simultaneously, the intermediate culture was gradually cooled to 12°C and the calculated inoculum volumes were mixed with fresh 12 ^o^P wort to a final 200 ml volume with 0.1 ml L^−1^ autoclaved Antifoam A reagent (Sigma-Aldrich, Germany) in 300 ml infusion bottles with a screw cap and septum (Avantor™, Netherlands) and a 3 cm stirring magnet. Release of CO_2_ and pressure were controlled *via* a Microlance needle (Fisher Scientific, UK) covered with cotton to decrease contaminations. Fermentations were carried out at 12°C and the fermentation progress was monitored measuring weight loss through time. Samples were taken with a Microlance needle (Fisher Scientific, UK) and 5 ml were sampled for HPLC measurements of ethanol, glucose, maltose, and maltotriose.

Green beer was analyzed *via* gas chromatography /mass spectrometry (GC/MS) as described in Rollero et al. (2015) to determine concentrations of volatile compounds of interest. Aroma compound concentrations [mg L^−1^] were derived from triplicate biological fermentations with high reproducibility including values below and above the sensitivity of the GC/MS analysis per compound listed in Supplementary Table S10. Significant differences between each compound were calculated (*p*-values <0.05) with one-way ANOVA and Tukey’s multiple comparisons test (Supplementary Table S11). Z-scores were calculated per aroma compound by subtracting the mean of the sample from the observed value, divided by the standard deviation of the sample.

## Results

### HTS of *Saccharomyces* strains identified *Saccharomyces mikatae* strains exhibiting growth on maltotriose

The ability to utilize maltotriose is typically associated with ale *S. cerevisiae* and *S. pastorianus* strains, while limited utilization is seen throughout non-domesticated yeast species (Methner et al., 2019; Nikulin et al., 2020a). Nevertheless, relatively few yeast strains have been tested for this phenotype due to lack of appropriate screening tools and the high cost of the oligosaccharide in its pure form. Here, a previously described high-throughput assay for phenotyping microorganisms on solid media - PHENOS (Barton et al., 2018) was adopted to identify non-*cerevisiae Saccharomyces* strains with the ability to grow on mineral medium with maltotriose as the sole carbon source. Nearly 200 *Saccharomyces* wild-type strains from various species, geographical locations, and origins were selected for the analysis and were initially tested on solid YP + Mtt at 30°C (Supplementary Figure S1) in two arrays – I and II (Supplementary Table S1). The strains tested comprise all eight currently known species of the *Saccharomyces* clade (except for *S. jurei*) and the domesticated *Saccharomyces* hybrids *S. pastorianus* and *Saccharomyces bayanus*. Well-characterized control strains with known maltotriose phenotypes were included, i.e., *S. pastorianus* CBS 1513 (Mtt^+^) and *S. eubayanus* CBS 12357^T^ (Mtt^−^). The Mtt^+^ phenotype observed for the Mtt^−^ control strain indicated that the medium required optimization (Supplementary Figure S1). Therefore, synthetic medium with maltotriose (SM + Mtt) and a different maltotriose batch with a higher purity were used in a next step to test the best performers in a single array including two *S. jurei* strains (Supplementary Table S1: Array III). Performance was ranked based on maximum slope values generated *via* the PHENOS software (equivalent μ_max_). The value for the strain *S. eubayanus* strain CBS 12357^T^ (Mtt^−^) was set to 1 and the performances of all other strains were shown as fold-change in comparison (Supplementary Figure S2B). The top-performing strains included four non-*cerevisiae Saccharomyces* strains—the *S. jurei* D5095^T^ and *S. mikatae* LSYS65-1 strains which were recently reported as Mtt^+^ in parallel studies (Giannakou et al., 2021; Peris et al., 2022) and two *S. mikatae* strains not described yet in literature for the phenotype tested—NBRC 11002 and NBRC 10997 (Supplementary Figure S2).

**Figure 1:**
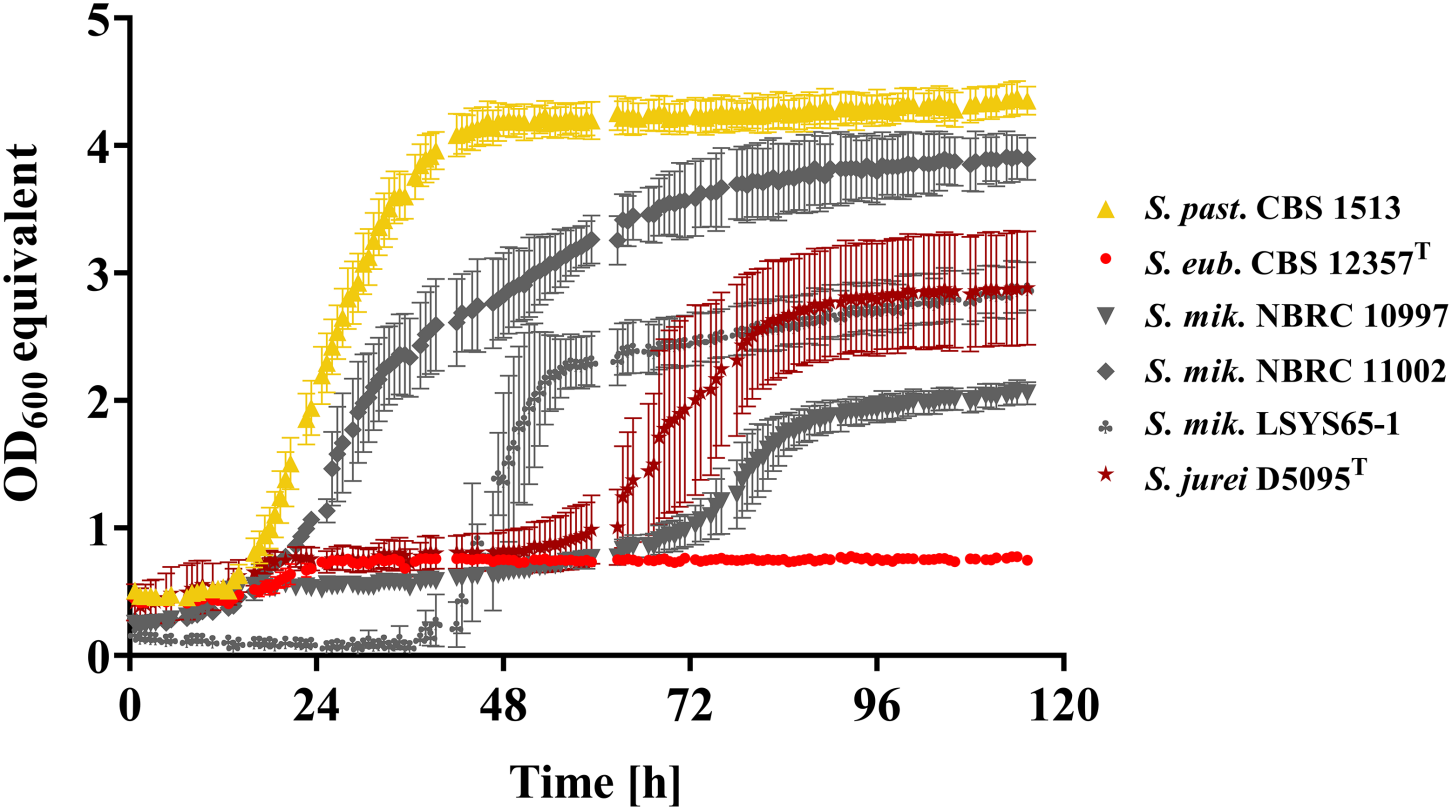
Growth kinetics in synthetic medium containing 2% (w/v) maltotriose (Mtt) of *S. pastorianus* CBS 1513 (Mtt^+^; ▲), *S. eubayanus* CBS 12357^T^ (Mtt^−^; ●) and the non-*S. cerevisiae* maltotriose-positive candidates: *S. jurei* D5095^T^ (★), *S. mik*atae LSYS65-1 (♣), *S. mik*atae NBRC 11002 (♦) and *S. mikatae* NBRC 10997 (▼) recorded *via* Growth Profiler 1,152. Values are presented as mean and standard deviation of biological triplicates. Raw data are provided in Supplementary Table S3.

To eliminate any potential impurities in the components used in our solid medium (i.e., agar) that could have caused the residual growth of Mtt^−^ strains, all superior Mtt^+^ candidate strains and our Mtt^−^ and Mtt^+^ control strains were further analyzed in liquid SM + Mtt. To this end, the phenotyping was conducted in small volumes using the Growth Profiler. Under the respective conditions, only minimal growth was observed for CBS 12357^T^ (Mtt^−^) contrasting the well-performing Mtt^+^control, i.e., the strain *S. pastorianus* CBS 1513 (Figure 1). Real quantitative data such as maximum specific growth rates and lag phase durations could not be presented for the newly discovered non-domesticated Mtt*^+^* candidates due to flocculation of all four strains in SM. Nevertheless, all strains successfully grew on maltotriose as the sole carbon source. Three of the four superior Mtt*^+^* strains exhibited a long lag phase while *S. mikatae* NBRC 11002 interestingly switched metabolism from glucose (pre-culture) to maltotriose in SM without much delay similar to *S. pastorianus* CBS 1513 (Figure 1).

### Hybridizing *Saccharomyces eubayanus* CBS 12357^T^ (Mtt^−^) with *Saccharomyces mikatae* NBRC 10997 (Mtt^+^) or *Saccharomyces jurei* D5095^T^ (Mtt^+^) resulted in a drastically improved aerobic growth on maltotriose due to heterosis

It was decided to combine the maltotriose utilization capabilities of the newly identified non-*cerevisiae Saccharomyces* Mtt^+^ candidates with the Mtt^−^ but cold-tolerant *S. eubayanus* strain CBS 12357^T^ in a hybrid and check for the heterosis effect in a comparative growth experiment in liquid medium. To this end, it was intended to introduce auxotrophic markers in the parental strains; however, complementary auxotrophs to facilitate mating by complementation were not successfully isolated. Instead, it was decided to rapidly generate stable haploids *via* genetically engineering the HO endonuclease to abolish self-mating (Strathern et al., 1982). This approach allows the generation of *MATa*/*MATα ho*/*ho* allodiploids which could be further exploited for future quantitative genetic studies on multiple generations of random mating of allotetraploid hybrids with restored fertility (Naseeb et al., 2021). Subsequently, *de novo* hybrids between *S. eubayanus* CBS 12357^T^ and *S. jurei* D5095^T^ and between *S. eubayanus* CBS 12357^T^ and *S. mikatae* NBRC 10997, i.e., NG92 (*Se* × *Sj*) and NG101 (*Se* × *Sm*) were successfully constructed.

**Figure 2:**
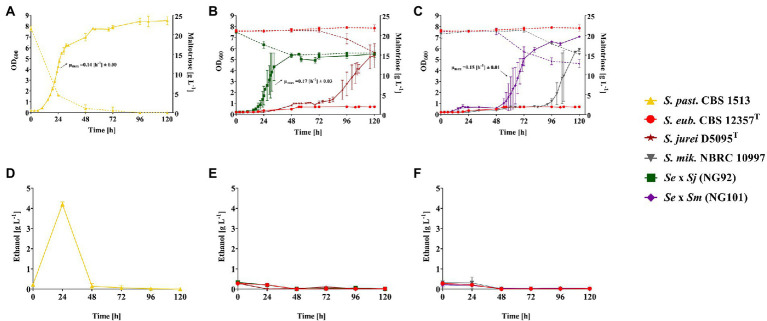
Growth in liquid synthetic medium with 2% (w/v) maltotriose (Mtt) of strains *S. pastorianus* CBS 1513 (Mtt^+^; ▲), *S. eubayanus* CBS 12357^T^ (Mtt^−^; ●), *S. jurei* D5095^T^ (Mtt^+^; ★), *S. mikatae* NBRC 10997 (Mtt^+^; ▼) and the constructed *de novo* hybrids – NG92 (*S. eubayanus* CBS 12357^T^ × *S. jurei* D5095^T^/*Se* × *Sj*; ■) and NG101 (*S. eubayanus* CBS 12357^T^ × *S. mikatae* NBRC 10997/*Se* × *Sm*; ♦). Values are presented as mean and standard deviation of triplicate biological replicates. **(A–C)** Growth curves depicting OD_600_ values and maltotriose consumption [g L^−1^] illustrated with continuous and dotted lines, respectively. Maximum specific growth rates (μ_max_ [h^−1^]) were calculated for all hybrids but not for the corresponding parental strains due to flocculation. **(D–F)** Ethanol production [g L^−1^] measured every 24 h. Raw data are provided in Supplementary Table S7.

To record the kinetics of sugar consumption and ethanol formation in addition to biomass formation of the strains during aerobic growth in liquid SM + Mtt, a higher culture volume was required for frequent sampling of culture supernatants. Thus, the previous Growth Profiler experimental setup (Figure 1) was upscaled to 50 ml cultures in shake flasks which were inoculated with the *de novo* hybrids - NG92 (*Se* × *Sj*) and NG101 (*Se* × *Sm*) in parallel with the corresponding parental strains and the *S. pastorianus* strain CBS 1513 as Mtt^+^ reference. As expected, the *S. pastorianus* strain CBS 1513 completed maltotriose consumption after 48 h of cultivation in SM + Mtt (Figure 2A). In contrast, the hybrids NG92 (*Se* × *Sj*) and NG101 (*Se* × *Sm*) only consumed 25 and 35%, of sugar, respectively, after 120 h of cultivation (Figures 2B,C). Interestingly, the two hybrids did not flocculate in comparison to their respective *S. mikatae*/*S. jurei* parental strain allowing growth rate determination during exponential phase. Remarkably, the two *de novo* hybrids outperformed both parental strains exhibiting growth rates [h^−1^] comparable to the *S. pastorianus* strain CBS 1513 and a shorter lag phase [h] compared to the parental Mtt^+^ strains *S. jurei* D5095^T^ and *S. mikatae* NBRC 10997 (Figures 2A–C). NG92 (*Se* × *Sj*) displayed a significantly shorter lag phase than NG101 (*Se* × *Sm*; Figures 2B,C).

Notably, the *S. pastorianus* strain CBS 1513 produced ethanol from maltotriose under the applied conditions. After 24 h of cultivation, an ethanol concentration of 4 g L^−1^ was detected (Figure 2D). The fact that there was no remaining ethanol after 48 h demonstrated that the ethanol formed was afterward consumed. However, no ethanol formation could be detected in either of the two *de novo* hybrids NG92 (*Se* × *Sj*) and NG101 (*Se* × *Sm*; Figures 2E,F). Probably, these strains indeed did not show any overflow metabolism under the tested conditions or the appropriate time point representing the ethanol production peak was simply missed during sampling.

### The *de novo* hybrids NG92 (*Se* × *Sj*) and NG101 (*Se* × *Sm*) completely fermented maltose, in contrast to their corresponding *Saccharomyces jurei*/*Saccharomyces mikatae* parents, but not maltotriose in lager brewing conditions

**Figure 3:**
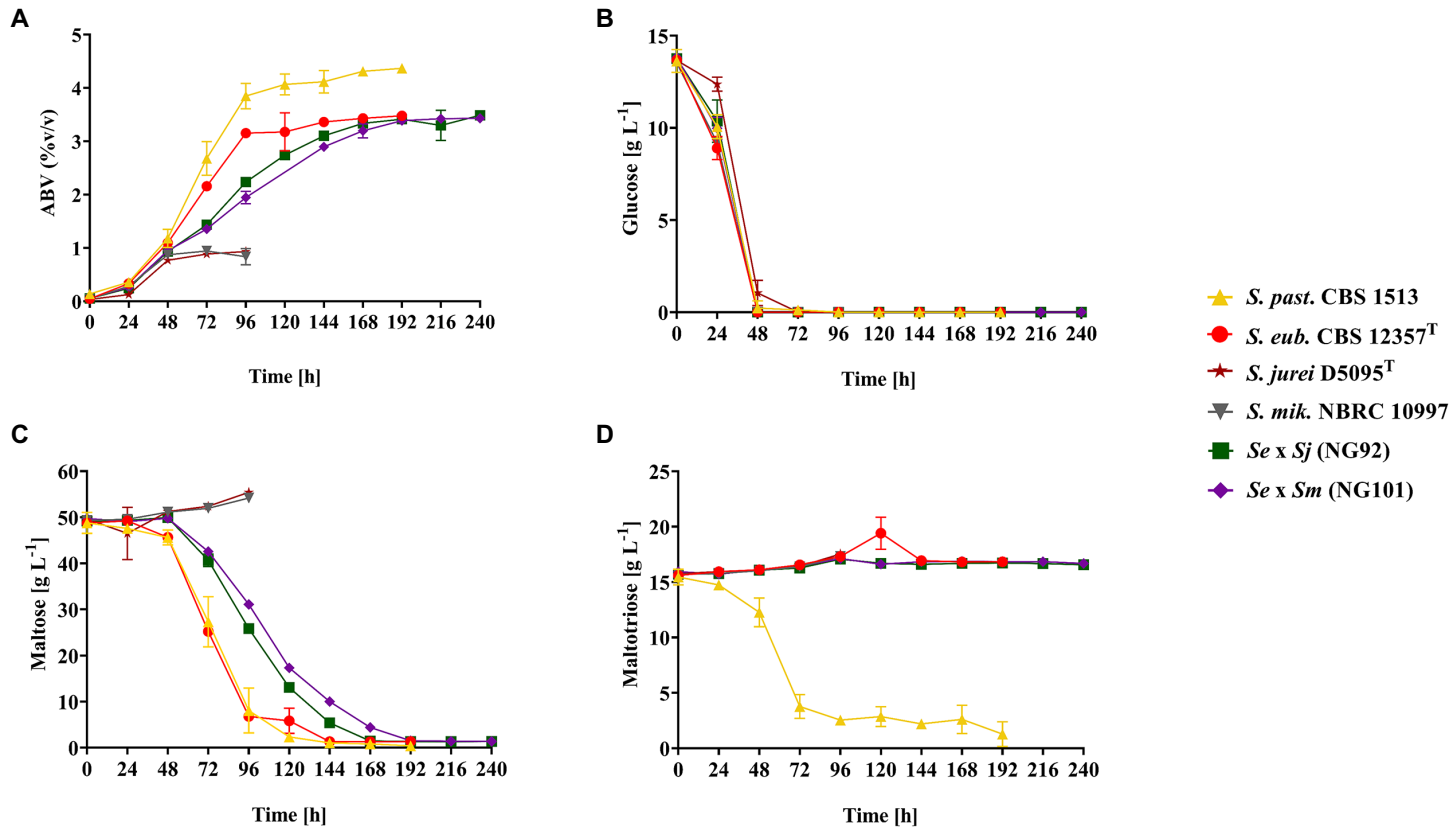
Fermentation in oxygen-limited conditions monitored by ethanol formation represented as alcohol by volume (ABV) [%] at 12°C for 10 days of fermentation of 12 ^o^P wort. The strains tested include the maltotriose-positive (Mtt^+^) strain *S. pastorianus* CBS 1513 (▲) as lager reference, the parental strains – *S. eubayanus* CBS 12357^T^ [maltotriose-negative (Mtt^−^)] (●), *S. jurei* D5095^T^ (Mtt^+^; ★), *S. mikatae* NBRC 10997 (Mtt^+^; ▼) and the constructed *de novo* hybrids - NG92 (*S. eubayanus* CBS 12357^T^ × *S. jurei* D5095^T^*/Se* × *Sj*; ■) and NG101 (*S. eubayanus* CBS 12357^T^ × *S. mikatae* NBRC 10997/*Se* × *Sm*; ♦). Reduced oxygen availability was achieved by using relatively high culture volumes (200 ml in 300 ml infusion bottles). The bottles were equipped with a screw cap and septum with a needle allowing the release of CO_2_ with reduced shaking frequency. **(A)** Fermentation progress, **(B)** Glucose consumption, **(C)** Maltose consumption and **(D)** Maltotriose consumption [g L^−1^] measured every 24 h. Values are presented as mean and standard deviation of triplicate biological fermentations. Raw data are provided in Supplementary Table S8.

Eventually, it was interesting to test the novel hybrids under conditions which are relevant in lager brewing. To this end, brewer’s wort fermentations at 12°C were conducted in lab scale with the constructed *de novo* hybrids—NG92 (*Se* × *Sj*) and NG101 (*Se* × *Sm*), all parental strains—*S. eubayanus* CBS 12357^T^, *S. jurei* D5095^T^, and *S. mikatae* NBRC 10997 as well as *S. pastorianus* CBS 1513. The latter strain was added as a reference since it is regularly used in industrial lager beer production (Figure 3). Within the first 48 h, all strains rapidly fermented glucose (Figure 3B) including the residual amounts of fructose and sucrose estimated by the calculated total sugar consumption *via* weight/CO_2_ loss measurements (Supplementary Table S8). After this time point, *S. pastorianus* CBS 1513 and *S. eubayanus* CBS 12357^T^, NG92 (*Se* × *Sj*), and NG101 (*Se* × *Sm*) completely utilized maltose without an apparent lag phase, while the parental *S. jurei* D5095^T^ and *S. mikatae* NBRC 10997 strains did not utilize the disaccharide at all. Notably, the *de novo* hybrids exhibited a slower rate of maltose fermentation compared to *S. pastorianus* CBS 1513 and *S. eubayanus* CBS 12357^T^ and required additional 24–48 h to complete the utilization of the sugar (Figure 3C). As expected, the domesticated lager hybrid (*S. pastorianus*) consumed maltose most rapidly and ethanol formation of 4.4% alcohol by volume (ABV) was achieved. Moreover, *S. pastorianus* CBS 1513 co-consumed maltose and maltotriose once glucose was depleted as previously described (Magalhães et al., 2016; Figures 3C,D). The two hybrids, NG92 (*Se* × *Sj*) and NG101 (*Se* × *Sm*), could not utilize maltotriose during the conducted wort fermentations (Figure 3D) similar to *S. eubayanus* CBS 12357^T^ only reaching an ABV of ~3.5% (Figure 3A) although they efficiently grew on maltotriose as the sole carbon source in the shake-flask cultivations at 25°C, with comparable maximum specific growth rates to *S. pastorianus* CBS 1513 (Figures 2A–C).

### Green beer produced by *de novo* hybrids NG92 (*Se* × *Sj*) and NG101 (*Se* × *Sm*) showed increased concentrations of fruity flavor esters

**Figure 4:**
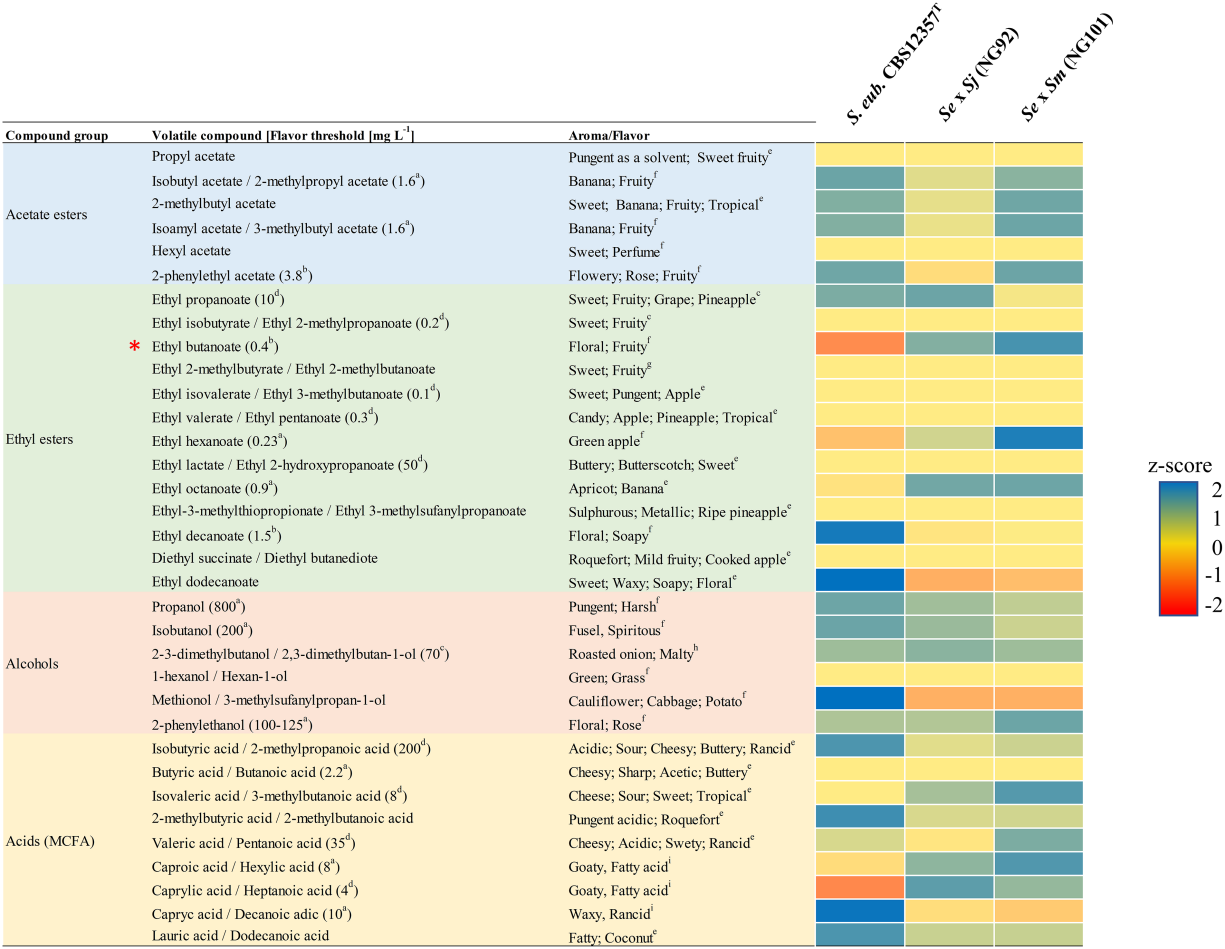
Comparative analysis of aroma production by the generated *de novo* hybrids NG92 (*S. eubayanus* CBS 12357^T^ × *S. jurei* D5095^T^/*Se* × *Sj*) and NG101 (*S. eubayanus* CBS 12357^T^ × *S. mikatae* NBRC 10997/*Se* × *Sm*) and their common parental strain *S. eubayanus* CBS 12357^T^. Colors show the range of the calculated *Z*-scores per aroma compound indicating production values above average, average, and below average shown with blue, yellow, and red, respectively. The aroma compounds are grouped by acetate esters, ethyl esters, alcohols, and acids (medium-chain fatty acids/MCFA). Flavor thresholds in beer values (shown in brackets) were obtained from Meilgaard (1982) (^a^); Meilgaard (1975a) (^b^); Meilgaard (1975b) (^c^); Harrison (1970) (^d^). Corresponding aromas/flavors for each volatile compound tested were obtained from The Good Scent Company Information System (2022) (^e^); Swiegers et al. (2005) (^f^); PubChem (2022) (^g^); Dunlevy et al. (2009) (^h^); Blanco et al. (2016) (^i^). The calculations are based on aroma compound concentrations [mg L^−1^] from triplicate biological fermentations with high reproducibility including values below and above the sensitivity of the GC/MS analysis. Detailed quantitative data of the aroma compounds with standard deviation of all strains including the aroma profile of the reference *S. pastorianus* CBS 1513 strain are presented in Supplementary Table S9. Z-scores generated from concentrations below or just above the threshold of measurement are marked with an asterisk (*).

The green lager beers produced by the *de novo* hybrids NG92 (*Se* × *Sj*) and NG101 (*Se* × *Sm*) as well as their respective *S. jurei*/*S. mikatae* parent and the common parent *S. eubayanus* CBS 12357^T^ (Figure 3) were comparatively analyzed with GC/MS for their aroma profiles. In addition, the lager strain *S. pastorianus* CBS 1513 was used as a reference. Green beers obtained with the *de novo* hybrids showed different concentrations of aroma compounds compared to their parental strains (Supplementary Table S9). In fact, heterotic production of desired esters was achieved, especially for NG101 (*Se* × *Sm*; Supplementary Table S9). We compare here the actual aroma concentration values detected keeping in mind that the parental strains – *S. jurei* D5095^T^ and *S. mikatae* NBRC 10997 could not ferment maltose and maltotriose, therefore, they produced concentrations close or below the lower threshold of measurement. Accurate comparison between the *S. eubayanus* strain CBS 12357^T^ and the interspecific hybrids is possible reaching an equal final ABV of 3.4–3.5% (Figure 4). Notably, enhanced production of ethyl esters such as ethyl hexanoate (green apple) was observed for NG92 (*Se* × *Sj*) which was 2.5-fold higher in comparison to the *S. eubayanus* CBS 12357^T^ parent, while NG101 (*Se* × *Sm*) produced this aroma compound in amounts above its flavor threshold in lager beer (Figure 4). Moreover, ethyl octanoate (apricot) concentration levels were ~2-fold higher for both hybrids compared to CBS 12357^T^ (Figure 4). Comparable concentrations were detected for different acetate esters tested between NG92 (*Se* × *Sj*), NG101 (*Se* × *Sm*), and CBS 12357^T^.

Interestingly, higher concentrations of isoamyl acetate (banana; 5.22 mg L^−1^) above the reported flavor threshold in beer and 2-phenylethyl acetate (3.75 mg L^−1^) close to its threshold of perception were determined for the NG101 (*Se* × *Sm*) hybrid compared to the industrial lager hybrid – *S. pastorianus* CBS 1513 (Supplementary Table S9). Similar amounts were identified between all hybrids and *S. eubayanus* CBS 12357^T^ for favorable higher alcohols such as 2-phenylethanol (rose, floral) and propanol (spicy, hard). However, the off-flavor 2,3-dimethylbutan-1-ol (roasted onion) associated typically with wine fermentations was produced with a slightly higher concentration in the *de novo* hybrids (Supplementary Table S9). Undesired medium-chain fatty acids (MCFA) were detected for all strains tested with significantly higher concentrations of caprylic acid (goaty) produced by *S. pastorianus* CBS 1513 albeit below its taste threshold (Supplementary Table S9). Although we present here a diverse set of aroma compounds with interesting heterotic production of desired fruity esters, the combined aroma profile could not be assessed in a sensory analysis due to the use of genetic engineering to construct the strains.

## Discussion

The use of wild yeasts and *de novo* hybrids in brewing is an attractive approach toward diversifying the lager beer style. However, the challenge is to meet pleasant taste with fermentation performance at the relative cold fermentation temperatures during lager beer brewing. The consumption of the wort sugar maltotriose is an important trait; however, non-conventional *Saccharomyces* strains able to utilize maltotriose have not been reported at the time when the current study was initiated.

Our HTS identified so far uncharacterized *S. mikatae* Mtt^+^ wild strains as well as the recently reported Mtt^+^ *S. mikatae* LSYS65-1 strain (Peris et al., 2022). Parallel studies conducted by other authors demonstrated that the two *S. jurei* strains TUM 629 and D5095^T^ are also able to utilize wort maltotriose to a certain extent (Giannakou et al., 2021; Hutzler et al., 2021) and that some *S. mikatae* strains are able to grow on this sugar (Peris et al., 2022). The current study included the strain *S. jurei* D5095^T^ and confirmed its Mtt^+^ phenotype. The *S. jurei* D5095^T^ (Mtt^+^) strain and one of the identified Mtt^+^ *S. mikatae* strains in this study (NBRC 10997) were used to construct novel hybrids with the *S. eubayanus* strain CBS 12357^T^. We demonstrated several clear heterosis effects in the hybrids as discussed below.

Our HTS of ~200 strains of the clade *Saccharomyces* for growth on maltotriose as the sole source of carbon revealed limitations when conducted on solid medium. In fact, the negative control strain *S. eubayanus* CBS 12357^T^ showed significant growth in the PHENOS phenotyping. Still, the latter method successfully identified superior Mtt^+^ isolates of non-domesticated yeasts. Their Mtt^+^ phenotypes were afterward confirmed in a liquid medium *via* the Growth Profiler.

Our finding that *S. mikatae* strains exhibit growth on maltotriose as the sole carbon source provides an example of the high intraspecies phenotypic diversity uncovered recently for the species despite its low genetic diversity (Peris et al., 2022). However, only a limited number of strains have been tested so far for wort fermentability. In fact, Nikulin et al. (2018) showed that neither α-glucoside (maltose and maltotriose) in brewer’s wort was utilized by the *S. mikatae* type strain (IFO 1815). Moreover, authors only tested the strain’s performance at 12°C which, in the current study, turned out to be unfavorable for the utilization of the respective wort sugars by *S. mikatae*.

So far, *S. mikatae* strains have only been isolated in East Asia even though evidence of the species’ presence in Europe was provided by sequencing the ITS1 region from DNA obtained from the mycobiome of soils surrounding trees in the Italian alps (Alsammar et al., 2019). Interestingly, *S. jurei* is the most closely related species to *S. mikatae* (Naseeb et al., 2018) and strains of *S. jurei* have already been isolated in Europe. The existence of Mtt^+^ isolates in both species seems to be in contrast to all other non-domesticated *Saccharomyces* species. Although more data are required to verify this, one might hypothesize that the ability to grow on maltotriose could have been obtained before the evolutionary split of the two species.

Phylogenetic distribution of *MAL* genes throughout the *Saccharomyces* clade suggests that maltotriose transport is the limiting factor (Day et al., 2002; Baker and Hittinger, 2019), mediated by Agt1 (Alves et al., 2008) and Mtt1 (Dietvorst et al., 2005), while certain diastatic yeasts strains can degrade the sugar extracellularly prior to transport (Alves et al., 2018). Hutzler et al. (2021) confirmed maltotriose transmembrane transport in the *S. jurei* strain TUM 629 employing radiolabeled substrate. The authors conducted a genetic analysis on the respective strain and identified a gene with 82.6% sequence identity to *S. cerevisiae AGT1*, which can potentially encode for a maltotriose permease. This might also apply to *S. jurei* D5095^T^, considering the low sequence divergence of the two strains (0.1%). The three new Mtt^+^ *S. mikatae* strains identified here (LSYS65-1, NBRC 11002, and NBRC 10997) are interesting candidates to further study the genetic basis underlying the Mtt^+^ phenotypes in the species *S. jurei* and *S. mikatae*. For whole-genome sequencing, it is recommended to use third-generation sequencing to properly assemble sub-telomeric regions which cannot be dissected by Illumina sequencing. As known from other Mtt^+^ strains of the *Saccharomyces* clade, the sub-telomeric regions usually contain the *MAL* genes.

One might further question whether the genetic resources causing the Mtt^+^ trait of the ale yeast *S. cerevisiae* can be replaced by the newly discovered Mtt^+^ wild strains (*S. jurei* and S. *mikatae*) when hybridized with the cold-tolerant *S. eubayanus* CBS 12357^T^. Enhancing traits relevant in brewing *via* interspecific *Saccharomyces* hybrids has been successfully applied in several previous studies (Hebly et al., 2015; Krogerus et al., 2015; Mertens et al., 2015; Nikulin et al., 2018; Giannakou et al., 2021). We hypothesized that heterosis regarding the Mtt^+^ phenotype could be achieved in such hybrids *via* synergistic actions of structural and regulatory proteins involved in maltotriose metabolism. Regarding the two sub-genomes of *S. pastorianus* (*S. cerevisiae* and *S. eubayanus*), a regulatory crosstalk between *MALR* and *MALT* genes has previously been demonstrated in a *de novo S. pastorianus* hybrid and provided a possible explanation for the efficient maltotriose fermentation of the domesticated lager yeast (Brouwers et al., 2019a). The common parent, *S. eubayanus* CBS 12357^T^, does not contain functional maltotriose transporters but has been shown to exhibit evolvability to the trisaccharide indicating the existence of genetic resources (Baker and Hittinger, 2019; Brouwers et al., 2019b). Interestingly, the *de novo* hybrids between Mtt^−^ *S. eubayanus* CBS 12357^T^ and Mtt^+^ *S. jurei* D5095^T^/*S. mikatae* NBRC 10997 successfully displayed heterosis with regard to their aerobic growth performance on maltotriose as the sole carbon source. Both hybrids, NG92 (*Se* × *Sj*) and NG101 (*Se* × *Sm*) outperformed the respective parents in terms of this trait, and both exhibited maximum specific growth rates comparable to the industrial *S. pastorianus* CBS 1513 strain. We presume that, in the *de novo* hybrids, an increased expression of *MALR* regulatory genes originating from the *S. eubayanus* CBS 12357^T^ genome could positively regulate potential transporter genes in the sub-genomes of *S. mikatae* NBRC 10997 and *S. jurei* D5095^T^. Notably, there was a significant difference in the lag phase duration between NG92 (*Se* × *Sj*) and NG101 (*Se* × *Sm*) upon this switch to maltotriose which might have been caused by differences in the sub-genome interactions between the common parent *S. eubayanus* CBS 12357^T^ and the *S. jurei* and *S. mikatae* parent, respectively.

Regardless of the remarkable maximum growth rates in SM + Mtt, it remains unknown why biomass formation of the *de novo* hybrids ended before maltotriose was depleted which was in clear contrast to *S. pastorianus* CBS 1513, which completely consumed the trisaccharide exhibiting a comparable growth rate. The latter partially assimilated maltotriose in a fermentative manner (as visible by ethanol production) in the aerobic shake-flask experiments. Simple shake-flask cultivations are known for limited oxygen supply. Moreover, no control over important variables such as pH is possible (Link and Weuster-Botz, 2011). Oxygen limitations might have resulted in a limited supply of ATP required to transport maltotriose through the cell membrane *via* the presumed maltotriose transporter in the two hybrids NG92 (*Se* × *Sj*) and NG101 (*Se* × *Sm*). Notably, the Agt1 permease has been shown to be an active maltotriose H^+^ symporter (Stambuk et al., 1999). A low substrate affinity of maltotriose transporters might contribute to the sudden growth termination of the *de novo* hybrids.

While aerobic growth on maltotriose as the sole carbon source is an interesting trait in the context of phylogeny, successful fermentation of the sugar to alcohol is of much greater importance for brewers. While strains of the domesticated species *S. pastorianus* strains are well known for the latter trait, the *de novo* hybrids NG92 (*Se* × *Sj*) and NG101 (*Se* × *Sm*) did not ferment maltotriose in spite of their ability to aerobically assimilate maltotriose. Nevertheless, the hybrids completely fermented maltose which contrasted with their corresponding *S. jurei*/ *S. mikatae* parents even though the rates were lower compared to that of the other co-parent, *S. eubayanus* CBS 12357^T^. An improvement of the wort sugar fermentation rates could be achieved *via* prior adaptation of the strains to wort. In fact, re-pitching in maltose-rich medium combined with cell viability monitoring were vital requirements to adapt *S. paradoxus* for wort fermentations (Nikulin et al., 2020b). Moreover, pre-adaptation to maltotriose was shown to be essential to achieve partial fermentation of the trisaccharide in wort by *S. jurei* TUM 629 (Hutzler et al., 2021). These findings suggest that further adaptive evolutionary experiments with NG92 (*Se* × *Sj*) and NG101 (*Se* × *Sm*) could be a promising approach to fully exploit their potential to ferment brewer’s wort. Nevertheless, the genome stability of the genetically engineered hybrids must be evaluated and directed evolution might be necessary to reach an industrial potential. The use of genetic modifications gave a convenient and rapid opportunity to study the fermentative and aromatic potential of such novel *de novo* hybrids in the lab environment. Still, alternative mating methods without genetic manipulations are strongly preferred if hybrids will be used in large-scale industrial applications in beer (Nakatomi and Gunge, 1972; Alexander et al., 2016; Gorter De Vries et al., 2019).

The most valuable traits of using wild strains and novel hybrids for beer consumers are the exotic aromas and flavors present in the beer produced with such yeast strains. In the hybrids constructed here with *S. eubayanus,* the parental wild *S. jurei* and *S. mikatae* strains contributed to the aroma profile. There was a heterotic production of desired fruity aroma compounds. Both NG92 (*Se* × *Sj*) and NG101 (*Se* × *Sm*) revealed favorable heterotic production of ethyl octanoate (apricot) and ethyl hexanoate (green apple). The latter was already outlined in the study of Hutzler et al. (2021) for contributing to the tropical final profile caused by *S. jurei* TUM 629 as detected *via* a sensory panel. Other desirable aromatic compounds in brewing such as 3-methylbutyl acetate (banana) and 2-phenylethyl acetate (rose, fruity) exhibited higher concentration in both hybrids compared to the commercial *S. pastorianus* CBS 1513 (Supplementary Table S9). The concentration of 2-phenylethanol was also increased in both *de novo* hybrids and increased concentrations of this compound have been highlighted to mask unwanted aroma compounds such as 4-VG notes typical for wild strains (Bamforth, 2020). The determination of important off-flavors such as diacetyl (butter-like; Duong et al., 2011) or 4-VG (clove-like; Coghe et al., 2004) was not in the scope of this study. Although the genetically modified organism (GMO)-nature of the constructed hybrids hampered any sensory evaluations, the measured concentrations of aroma compounds suppose a potential exotic tropical final aroma profile.

## Conclusion

The increase in the consumer demand for aromatic diversity in beer can be met in lager beer through interspecific hybridization of wild *Saccharomyces* yeast species. In such attempts, the utilization of maltotriose by the generated hybrids is a relevant trait for brewers. Here, we report maltotriose-utilizing strains from the species *S. mikatae* and confirmed the Mtt^+^ phenotype recently identified by others in strains of *S. jurei* (D5095^T^) and *S. mikatae* (LSYS65-1). We constructed two *de novo* hybrids: (i) between an Mtt^+^ *S. mikatae* strain isolated in the current study (NBRC 10997) and the Mtt^−^ but cold-tolerant *S. eubayanus* CBS 12357^T^ and (ii) between the Mtt^+^ *S. jurei strain* D5095^T^ and the *S. eubayanus* strain CBS 12357^T^. Both hybrids displayed heterosis on maltotriose and outperformed both parents regarding aerobic growth on maltotriose synthetic medium achieving growth rates comparable to the industrial reference strain *S. pastorianus* CBS 1513. In contrast to their *S. mikatae*/*S. jurei* parents, the *de novo* hybrids completely fermented maltose in wort fermentations in a lager brewing setting and produced enhanced amounts of favored fruity esters. Although the hybrids failed regarding the fermentation of maltotriose, there is the potential to improve this relevant trait as well as the rate of maltose fermentation by future adaptive laboratory evolution. The current study might therefore pave the way toward non-*cerevisiae Saccharomyces* hybrids able to completely ferment all wort sugars and brew exotic lager beers. By using complementary auxotrophic mutants and/or different physiological traits of the partners, non-GMO hybrids could be produced which could contribute to the diversification of lager beers in the future.

## Data availability statement

The original contributions presented in the study are included in the article/Supplementary material, further inquiries can be directed to the corresponding authors.

## Author contributions

NG conducted the experimental work and drafted the manuscript. ÁC-M, SW, and SA provided experimental assistance. EL and EN provided support in experimental setup and writing guidance. NK provided support in experimental setup and critical revision of the manuscript. All authors contributed to the article and approved the submitted version.

## Funding

The project was funded by the European Union’s Horizon 2020 Research and Innovation Program under the Marie Skłodowska-Curie Grant Agreement No. 764927.

## Conflict of interest

NK was employed by HEINEKEN Supply Chain B.V.

The remaining authors declare that the research was conducted in the absence of any commercial or financial relationships that could be construed as a potential conflict of interest.

## Publisher’s note

All claims expressed in this article are solely those of the authors and do not necessarily represent those of their affiliated organizations, or those of the publisher, the editors and the reviewers. Any product that may be evaluated in this article, or claim that may be made by its manufacturer, is not guaranteed or endorsed by the publisher.
