# Supplementary material for: Beyond Saccharomyces pastorianus for modern lager brews: Exploring non-cerevisiae Saccharomyces hybrids with heterotic maltotriose consumption and novel aroma profile
Source: Front Microbiol. 2022 Nov 10;13:1025132. doi: 10.3389/fmicb.2022.1025132 (PMC9687090; doi:10.3389/fmicb.2022.1025132)
Supplement: Supplementary file 9 [file Table_9.PDF]

Compound group	Volatile compound [Flavor threshold [mg L^{-1}]	Aroma/Flavor	*S. past.* CBS 1513	*S. mik.* NBRC 10997	*S. jurei* D5095T	*S. eub.* CBS12357T	*Se x Sj* (NG92)	*Se x Sm* (NG101)
			Aroma concentraion [mg L^{-1}]					
Acetate esters	Propyl acetate	Pungent as a solvent; Sweet fruity[e]	0.00	0.00	0.00	0.00	0.00	0.00
	Isobutyl acetate / 2-methylpropyl acetate (1.6[a])	Banana; Fruity[f]	0.15 ± 0.01	0.00	0.00	0.23 ± 0.01	0.14 ± 0.01	0.21 ± 0.02
	2-methylbutyl acetate	Sweet; Banana; Fruity; Tropical[e]	0.71 ± 0.06	0.03 ± 0.01	0.03 ± 0.01	1.01 ± 0.03	0.65 ± 0.03	1.07 ± 0.11
	Isoamyl acetate / 3-methylbutyl acetate (1.6[a])	Banana; Fruity[f]	3.28 ± 0.21	0.34 ± 0.05	0.36 ± 0.06	4.86 ± 0.13	3.15 ± 0.10	5.22 ± 0.51
	Hexyl acetate	Sweet; Perfume[f]	0.00	0.00	0.00	0.00	0.00	0.00
	2-phenylethyl acetate (3.8[b])	Flowery; Rose; Fruity[f]	1.87 ± 0.28	0.23 ± 0.01	0.18 ± 0.02	3.71 ± 0.31	1.72 ± 0.08	3.75 ± 0.23
Ethyl esters	Ethyl propanoate (10[d])	Sweet; Fruity; Grape; Pineapple[e]	0.19 ± 0.02	0.00	0.00	0.17 ± 0.01	0.18 ± 0.01	0.10 ± 0.01
	Ethyl isobutyrate / Ethyl 2-methylpropanoate (0.2[d])	Sweet; Fruity[e]	0.00	0.00	0.00	0.00	0.00	0.00
	Ethyl butanoate (0.4[b])	Floral; Fruity[f]	0.03 ± 0.00	0.00	0.00	0.00	0.04 ± 0.01	0.05 ± 0.01
	Ethyl 2-methylbutyrate / Ethyl 2-methylbutanoate	Sweet; Fruity[g]	0.00	0.00	0.00	0.00	0.00	0.00
	Ethyl isovalerate / Ethyl 3-methylbutanoate (0.1[d])	Sweet; Pungent; Apple[e]	0.00	0.00	0.00	0.00	0.00	0.00 ± 0.11
	Ethyl valerate / Ethyl pentanoate (0.3[d])	Candy; Apple; Pineapple; Tropical[e]	0.00	0.00	0.00	0.00	0.00	0.00
	Ethyl hexanoate (0.23[a])	Green apple[f]	0.19 ± 0.00	0.00	0.00	0.06 ± 0.01	0.15 ± 0.02	0.31 ± 0.04
	Ethyl lactate / Ethyl 2-hydroxypropanoate (50[d])	Buttery; Butterscotch; Sweet[e]	0.00	0.00	0.00	0.00	0.00	0.00
	Ethyl octanoate (0.9[a])	Apricot; Banana[e]	1.61 ± 0.16	0.06 ± 0.01	0.14 ± 0.02	0.38 ± 0.02	0.71 ± 0.05	0.72 ± 0.11
	Ethyl-3-methylthiopropionate / Ethyl 3-methylsufanylpropanoate	Sulphurous; Metallic; Ripe pineapple[e]	0.00	0.00	0.00	0.00	0.00	0.00
	Ethyl decanoate (1.5[b])	Floral; Soapy[f]	0.76 ± 0.03	0.00	0.00	0.58 ± 0.02	0.18 ± 0.01	0.19 ± 0.00
	Diethyl succinate / Diethyl butanediote	Roquefort; Mild fruity; Cooked apple[e]	0.00	0.00	0.00	0.00	0.00	0.00
	Ethyl dodecanoate	Sweet; Waxy; Soapy; Floral[e]	0.20 ± 0.02	0.02 ± 0.00	0.02 ± 0.00	0.99 ± 0.04	0.04 ± 0.00	0.09 ± 0.00
Alcohols	Propanol (800[a])	Pungent; Harsh[f]	16.29 ± 0.53	5.81 ± 0.50	5.18 ± 0.42	14.87 ± 0.23	13.22 ± 0.56	12.26 ± 0.82
	Isobutanol (200[a])	Fusel, Spiritous[f]	57.89 ± 3.34	8.76 ± 1.14	6.79 ± 0.83	44.07 ± 0.60	38.37 ± 0.81	32.45 ± 1.68
	2-3-dimethylbutanol / 2,3-dimethylbutan-1-ol (70[c])	Roasted onion; Malty[h]	121.04 ± 2.84	70.41 ± 6.77	69.47 ± 5.83	167.94 ± 3.05	175.69 ± 8.44	167.96 ± 10.16
	1-hexanol / Hexan-1-ol	Green; Grass[f]	0.00	0.00	0.00	0.00	0.00	0.00
	Methionol / 3-methylsufanylpropan-1-ol	Cauliflower; Cabbage; Potato[f]	0.57 ± 0.01	0.00	0.00	0.44 ± 0.00	0.00	0.00
	2-phenylethanol (100-125[a])	Floral; Rose[f]	51.62 ± 3.57	26.60 ± 2.57	28.29 ± 1.81	56.71 ± 2.67	56.29 ± 5.40	64.92 ± 2.25
Acids (MCFA)	Isobutyric acid / 2-methylpropanoic acid (200[d])	Acidic; Sour; Cheesy; Buttery; Rancid[e]	0.98 ± 0.14	0.79 ± 0.09	0.50 ± 0.04	1.23 ± 0.05	0.95 ± 0.07	0.99 ± 0.05
	Butyric acid / Butanoic acid (2.2[a])	Cheesy; Sharp; Acetic; Buttery[e]	0.00	0.00	0.00	0.00	0.00	0.00
	Isovaleric acid / 3-methylbutanoic acid (8[d])	Cheese; Sour; Sweet; Tropical[e]	1.15 ± 0.18	1.16 ± 0.16	0.95 ± 0.03	1.24 ± 0.18	1.37 ± 0.03	1.49 ± 0.10
	2-methylbutyric acid / 2-methylbutanoic acid	Pungent acidic; Roquefort[e]	0.63 ± 0.11	0.49 ± 0.07	0.42 ± 0.02	1.10 ± 0.11	0.80 ± 0.04	0.82 ± 0.06
	Valeric acid / Pentanoic acid (35[d])	Cheesy; Acidic; Swety; Rancid[e]	0.11 ± 0.01	0.10 ± 0.01	0.09 ± 0.00	0.10 ± 0.00	0.09 ± 0.00	0.10 ± 0.00
	Caproic acid / Hexylic acid (8[a])	Goaty, Fatty acid[i]	1.95 ± 0.05	0.10 ± 0.00	0.26 ± 0.23	0.67 ± 0.03	1.20 ± 0.11	1.45 ± 0.13
	Caprylic acid / Heptanoic acid (4[d])	Goaty, Fatty acid[i]	6.78 ± 0.01	1.08 ± 0.07	2.18 ± 0.20	1.48 ± 0.14	3.00 ± 0.26	2.68 ± 0.28
	Capryc acid / Decanoic adic (10[a])	Waxy, Rancid[i]	1.05 ± 0.07	0.15 ± 0.01	0.20 ± 0.03	0.64 ± 0.07	0.29 ± 0.02	0.26 ± 0.01
	Lauric acid / Dodecanoic acid	Fatty; Coconut[e]	0.13 ± 0.01	0.00	0.00	0.18 ± 0.01	0.11 ± 0.00	0.11 ± 0.00
