# Supplementary material for: Beyond Saccharomyces pastorianus for modern lager brews: Exploring non-cerevisiae Saccharomyces hybrids with heterotic maltotriose consumption and novel aroma profile
Source: Front Microbiol. 2022 Nov 10;13:1025132. doi: 10.3389/fmicb.2022.1025132 (PMC9687090; doi:10.3389/fmicb.2022.1025132)
Supplement: Supplementary file 14 [file Data_Sheet_1.docx]

Supplementary Material

# Supplementary Figure Captions

Figure S1: Growth kinetics of ~ 200 *Saccharomyces* strains on solid YP medium with 2 % (w/v) maltotriose (Mtt) at 30 ^o^C generated with the PHENOS pipeline (Barton et al., 2018) represented as boxplots of (**A**) growth rate [h^-1^] (maximum slope), (**B**) adaptation time [h] (lag time) and (**C**) biomass production (maximum OD_600_ values). It has to be highlighted that the maltotriose-negative *S. eubayanus* strain CBS 12357^T^ exhibited growth in the conditions tested. Data points are split based on species/domesticated hybrids designations. The species/domesticated hybrids are labelled as follows: *S. pastorianus* (▲), (*S. bayanus*) (■), *S. cerevisiae* (◉), *S. eubayanus* (●), *S. mikatae* (▼), *S. kudriavzevii* (♦), *S. uvarum* (■), *S. paradoxus* (⬣), *S. arboricola* (★). Values are presented as mean from quadruplicate technical replicates. Raw data is provided in Tables S4 and S5.

Figure S2: Growth characteristics of 80 *Saccharomyces* strains generated in the PHENOS pipeline (Barton et al., 2018) on synthetic medium with 2 % (w/v) maltotriose (Mtt) at 25 ^o^C represented as boxplots of (**A**) growth rate [h^-1^] (maximum slope), (**C**) adaptation time [h] (lag time) and (**D**) biomass production (maximum OD_600_ values). Data points are split based on species designations. Data points of maltotriose-positive candidates based on growth rate values larger than the value of the maltotriose-negative (Mtt^-^) strain *S. eubayanus* strain CBS 12357^T^ are highlighted with a larger symbol with full color and separated by a red dash line in panel (A). (**B**) Growth performance on maltotriose ranked by growth rate [h-^1^] (maximum slope values). The value for the strain *S. eubayanus* strain CBS 12357^T^ (Mtt^-^) was set to 1 and the performances of all other strains were shown as fold-change in comparison. The species/domesticated hybrids are labelled as follows: *S. pastorianus* (▲), *S. cerevisiae* (◉), *S. eubayanus* (●), *S. mikatae* (▼), *S. kudriavzevii* (♦), *S. uvarum* (■), *S. paradoxus* (⬣), *S. arboricola* (★), *S. jurei* (★). Values are presented as a mean of quadruplicate technical replicates while valued for control strains maltotriose-positive (Mtt^+^) *S. pastorianus* CBS 1513 and *S. eubayanus* strain CBS 12357^T^ (Mtt^-^) are shown as mean of twenty-four replicates. Raw data is provided in Table S6.

# Supplementary Table caption for Table S9

Table S9: Quantitative data for the aroma production of the generated *de novo* hybrids and the corresponding parental strains: NG92 (*S. eubayanus* CBS 12357^T^ x *S. jurei* D5095^T^/*Se* x *Sj*), NG101 (*S. eubayanus* CBS 12357^T^ x *S. mikatae* NBRC 10997/*Se* x *Sm*), *S. eubayanus* CBS 12357^T^, *S. jurei* D5095^T^ and *S. mikatae* NBRC 10997 including the strain *S. pastorianus* CBS 1513 as a typical lager beer reference. The aroma compounds are categorized by acetate esters, ethyl esters, alcohols, and acids (medium-chain fatty acids (MCFA). The concentration differences per category are highlighted with increasing length of blue, green, orange, and yellow bars respectively. Flavor thresholds in beer values (shown in brackets) were obtained from Meilgaard, 1982 (^a^); Meilgaard, 1975a (^b^); (Meilgaard, 1975b (^c^); Harrison, 1970 (^d^). Corresponding aromas/flavors for each volatile compound tested were obtained from The Good Scent Company Information System, 2022 (^e^); Swiegers et al. (2005) (^f^); PubChem, 2022 (^g^); Dunlevy et al. (2009) (^h^); Blanco et al. (2016) (^i^). The calculations are based on aroma compound concentrations [mg L^-1^] from triplicate biological fermentations including values below and above the sensitivity of the GC/MS analysis. Statistical analysis shows significant difference for each aroma compound tested with P value < 0.05 (Table S11).

# Supplementary References

Barton, D. B. H., Georghiou, D., Dave, N., Alghamdi, M., Walsh, T. A., Louis, E. J., et al. (2018). PHENOS: A high-throughput and flexible tool for microorganism growth phenotyping on solid media. *BMC Microbiol.* 18. doi: 10.1186/s12866-017-1143-y.

Bensasson, D., Zarowiecki, M., Burt, A., and Koufopanou, V. (2008). Rapid evolution of yeast centromeres in the absence of drive. *Genetics* 178, 2161–2167. doi.org/10.1534/genetics.107.083980.

Bing, J., Han, P. J., Liu, W. Q., Wang, Q. M., and Bai, F. Y. (2014). Evidence for a Far East Asian origin of lager beer yeast. *Curr. Biology* 24, 380–381. doi.org/10.1016/j.cub.2014.04.031.

Blanco, C. A., Andrés-Iglesias, C., and Montero, O. (2016). Low-alcohol Beers: Flavor Compounds, Defects, and Improvement Strategies. *Crit. Rev. Food Sci. Nutr.* 56, 1379–1388. doi: 10.1080/10408398.2012.733979.

Cohen, J. D., Goldenthal, M. J., Chow, T., Buchferer, B., and Marmur, J. (1985). Organization of the MAL loci of Saccharomyces Physical identification and functional characterization of three genes at the MAL6 locus. *Mol. Gen. Genet.* 200, 1–8. doi: 10.1007/BF00383304.

Cubillos, F. A., Louis, E. J., and Liti, G. (2009). Generation of a large set of genetically tractable haploid and diploid Saccharomyces strains. *FEMS Yeast Res.* 9, 1217–1225. doi.org/10.1111/j.1567-1364.2009.00583.x.

Dunlevy, J. D., Kalua, C. M., Keyzers, R. A., and Boss, P. K. (2009). Grapevine Molecular Physiology and Biotechnology*:* Second Edition (Springer, Dordrecht), 293–340.

Gerke, J. P., Chen, C. T. L., and Cohen, B. A. (2006). Natural isolates of Saccharomyces cerevisiae display complex genetic variation in sporulation efficiency. *Genetics* 174, 985–997. doi.org/10.1534/genetics.106.058453.

Goldstein A. L., and McCusker J. H. (1999). Three new dominant drug resistance cassettes for gene disruption in Saccharomyces cerevisiae. *Yeast* 15, 1541–1553. doi:10.1002/(SICI)10970061(199910)15:14<1541::AID-YEA476>3.0.CO;2-K.

Gonçalves, P., Valério, E., Correia, C., de Almeida, J. M. G. C. F., and Sampaio, J. P. (2011). Evidence for divergent evolution of growth temperature preference in sympatric saccharomyces species. *PLoS ONE* 6. doi.org/10.1371/journal.pone.0020739.

Harrison, G. A. F. (1970). The flavour of beer – a review. *Instr. Brew.* 76, 486–495. doi.org/10.1002/j.2050-0416.1970.tb03333.x

Hinks, A (2018). Saccharomyces hybrids: Generation and analysis [dissertation]. [Leicester, UK]: University of Leicester

Huxley C, Green E. D., Dunham I. (1990). Rapid assessment of S. cerevisiae mating type by PCR. *Trends Genet.* 6, 236. doi: 10.1016/0168-9525(90)90190-h.

Kim, H. S., and Fay, J. C. (2007). Genetic Variation in the Cysteine Biosynthesis Pathway Causes Sensitivity to Pharmacological Compounds. *PNAS USA* 104, 19387–19391. doi.org/10.1073/pnas.0708194104.

Leducq, J. B., Charron, G., Samani, P., Dubé, A. K., Sylvester, K., James, B, et al. (2014). Local climatic adaptation in a widespread microorganism. *Proc. Royal Soc. B.* 281. doi.org/10.1098/rspb.2013.2472.

Libkind, D., Hittinger, C. T., Valeŕio, E., Gonca̧lves, C., Dover, J., Johnston, M., et al. (2011). Microbe domestication and the identification of the wild genetic stock of lager-brewing yeast. *PNAS USA* 108, 14539–14544. doi.org/10.1073/pnas.1105430108.

Liti, G., Barton, D. B. H., and Louis, E. J. (2006). Sequence diversity, reproductive isolation and species concepts in saccharomyces. *Genetics* 174, 839–850. doi.org/10.1534/genetics.106.062166.

Liti, G., Carter, D. M., Moses, A. M., Warringer, J., Parts, L., James, S. A., et. al. (2009). Population genomics of domestic and wild yeasts. *Nature* 458, 337–341. doi.org/10.1038/nature07743.

Lopes, C. A., Barrio, E., and Querol, A. (2010). Natural hybrids of S. cerevisiae × S. kudriavzevii share alleles with European wild populations of Saccharomyces kudriavzevii. *FEMS Yeast Res.* 10, 412–421. doi.org/10.1111/j.1567-1364.2010.00614.x.

Meilgaard, M. C. (1975a). Flavor chemistry of beer: part II: flavor and threshold of 239 aroma volatiles. *Tech. Q. MBAA*, 12, 151–168.

Meilgaard, M. C. (1975b). Flavor chemistry of beer. I. flavor interaction between principal volatiles. *Tech. Q. MBAA*, 12, 107–117.

Meilgaard, M. C. (1982). Prediction of Flavor Differences between Beers from Their Chemical Composition. *J. Agric. Food Chem.*, 30, 1009–1017. doi.org/10.1021/jf00114a002.

Masneuf-Pomarede, I., Salin, F., Börlin, M., Coton, E., Coton, M., le Jeune, et al. (2016). Microsatellite analysis of Saccharomyces uvarum diversity. *FEMS Yeast Res.* 16*.* doi.org/10.1093/femsyr/fow002.

Muir A, Harrison E, and Wheals A (2011). A multiplex set of species-specific primers for rapid identification of members of the genus Saccharomyces. *FEMS Yeast Res.* 11, 552-563. doi.org/10.1111/j.1567-1364.2011.00745.x.

Naseeb, S., James, S. A., Alsammar, H., Michaels, C. J., Gini, B., Nueno-Palop, C., et al. (2017). Saccharomyces jurei sp. Nov., isolation and genetic identification of a novel yeast species from Quercus robur. *Int. J. Syst. Evol. Microbiol.* 67, 2046–2052. doi: 10.1099/ijsem.0.002013.

Naseeb, S., Visinoni, F., Hu, Y., Hinks Roberts, A. J., Maslowska, A., Walsh, T., et al. (2021). Restoring fertility in yeast hybrids: Breeding and quantitative genetics of beneficial traits. *PNAS* 118. doi.org/10.1073/pnas.2101242118/.

Naumov, G. I., James, S. A., Naumova, E. S., Louis, E. J., and Roberts, I. N. (2000). Three new species in the Saccharomyces sensu stricto complex : Saccharomyces cariocanus, Saccharomyces kudriavzevii and Saccharomyces mikatae. *Int. J.* *Syst. Evol. Microbiol.* 50, 1931–1942. doi.org/10.1099/00207713-50-5-1931.

Naumov, G. I., Naumova, E. S. and Louis, E. J (1995). Two new genetically isolated populations of the Saccharomyces sensu stricto complex from Japan. *J. Gen. Appl. Microbiol* 41, 499–505. doi.org/10.2323/JGAM.41.499.

Naumova E.S., Naumov G.I., and Molina F.I (2000). Genetic Variation Among European Strains of Saccharomyces paradoxus: Results from DNA Fingerprinting. *S. Appl. Microbiol.* 23, 86–92. doi.org/10.1016/S0723-2020(00)80049-1.

Nespolo, R. F., Villarroel, C. A., Oporto, C. I., Tapia, S. M., Vega-Macaya, F., Urbina, K. et al. (2020). An Out-of-Patagonia migration explains the worldwide diversity and distribution of Saccharomyces eubayanus lineages. *PLoS Genet.* *16*. doi.org/10.1371/journal.pgen.1008777.

Norrander J, Kempe T, and Messing J. (1983). Construction of improved M13 vectors using oligodeoxynucleotide-directed mutagenesis. *Gene* 26, 101–106. doi: 10.1016/0378-1119(83)90040-9.

Peris, D., Lopes, C. A., Arias, A., and Barrio, E. (2012). Reconstruction of the Evolutionary History of Saccharomyces cerevisiae x S. kudriavzevii Hybrids Based on Multilocus Sequence Analysis. *PLoS ONE* *7*. doi.org/10.1371/journal.pone.0045527.

Peter, J., de Chiara, M., Friedrich, A., Yue, J. X., Pflieger, D., Bergström, A. et al. (2018). Genome evolution across 1,011 Saccharomyces cerevisiae isolates. *Nature* 556, 339–344. doi.org/10.1038/s41586-018-0030-5.

Reuben, J., Pengelly, Wheals, and Alan, E. (2013). Rapid identification of Saccharomyces eubayanus and its hybrids. *FEMS Yeast Res.* 13, 156–161. doi.org/10.1111/1567-1364.12018.

PubChem (2022). https://pubchem.ncbi.nlm.nih.gov [Accessed March 12, 2022].

Sampaio, J. P., and Gonçalves, P. (2008). Natural populations of Saccharomyces kudriavzevii in Portugal are associated with Oak bark and are sympatric with S. cerevisiae and S. paradoxus. *Appl. Environ. Microbiol.* 74, 2144–2152. doi.org/10.1128/AEM.02396-07.

Sniegowski, P. D., Dombrowski, P. G., and Fingerman, E. (2002). Saccharomyces cerevisiae and Saccharomyces paradoxus coexist in a natural woodland site in North America and display different levels of reproductive isolation from European conspecifics. *FEMS Yeast Res.* 1, 299–306. doi.org/10.1111/j.1567-1364.2002.tb00048.x.

Stringini, M., Comitini, F., Taccari, M., and Ciani, M. (2009). Yeast diversity during tapping and fermentation of palm wine from Cameroon. *Food Microbiol.* 26, 415–420. doi.org/10.1016/j.fm.2009.02.006.

Swiegers, J. H., Bartowsky, E., and Pretorius, I. S. (2005). Yeast and bacterial modulation of wine aroma and flavour. *Aust. J. Grape Wine Res.* 11, 139–173. doi.org/10.1111/j.1755-0238.2005.tb00285.x.

The Good Scent Company Information System (2022). http://www.thegoodscentscompany.com [Accessed March 12, 2022].

van der Aa Kühle, A., Jesperen, L., Glover, R. L. K., Diawara, B., and Jakobsen, M (2001). Identification and characterization of Saccharomyces cerevisiae strains isolated from West African sorghum beer. *Yeast* 18, 1069–1079. doi.org/10.1002/yeast.756.

Wang, S. A., and Bai, F. Y. (2008). Saccharomyces arboricolus sp. nov., a yeast species from tree bark. *Int. J. Syst. Evol. Microbiol.* 58, 510–514. doi.org/10.1099/ijs.0.65331-0.

Wang, Q. M., Liu, W. Q., Liti, G., Wang, S. A. and Bai, F. Y. (2012). Surprisingly diverged populations of Saccharomyces cerevisiae in natural environments remote from human activity. *Mol. Ecol.* 21, 5404–5417. doi.org/10.1111/j.1365-294X.2012.05732.x.

White, T.J., Bruns, T.D., Lee, S.B. and Taylor, J.W. (1990) Amplification and Direct Sequencing of Fungal Ribosomal RNA Genes for Phylogenetics (*Academic Press*), 315-322Wimalasena, T. T., Greetham, D., Marvin, M. E., Liti, G., Chandelia, Y., Hart, A. et al. (2014). Phenotypic characterisation of Saccharomyces spp. yeast for tolerance to stresses encountered during fermentation of lignocellulosic residues to produce bioethanol. *Microb. Cell Factories* 13. doi.org/10.1186/1475-2859-13-47.

Zaki, A. M., Wimalasena, T. T., and Greetham, D. (2014). Phenotypic characterisation of Saccharomyces spp. for tolerance to 1-butanol. *J. Ind. Microbiol. Biotechnol.* 41, 1627–1636. doi.org/10.1007/s10295-014-1511-7.

Zhang, H., Richards, K. D., Wilson, S., Lee, S. A., Sheehan, H., Roncoroni, M. et al. (2015). Genetic characterization of strains of Saccharomyces uvarum from New Zealand wineries. *Food Microbiol.* 46, 92–99. doi.org/10.1016/j.fm.2014.07.016.
